# Appraisal of sexual functioning before and after cancer: insights from adolescent and young adult (AYA) cancer survivors and matched healthy controls

**DOI:** 10.1007/s00520-026-10925-2

**Published:** 2026-06-25

**Authors:** Chiara Acquati, Brenda L. den Oudsten, Stephanie Both, Vicky Lehmann

**Affiliations:** 1 Department of Family Medicine and Community Health, University of Minnesota, Minneapolis, MN USA; 2 Eli Coleman Institute for Sexual & Gender Health, University of Minnesota Medical School, Minneapolis, MN USA; 3 Graduate College of Social Work, University of Houston, 3511 Cullen Blvd, Houston, TX 77204-4013 USA; 4 Department of Health Disparities Research, The University of Texas MD Anderson Cancer Center, 1515 Holcombe Blvd, Houston, TX 77030 USA; 5 Department of Medical and Clinical Psychology, Tilburg School of Social and Behavioral Sciences, Tilburg University, Tilburg, The Netherlands; 6 Department of Sexology and Psychosomatic Gynecology and Obstetrics, Amsterdam University Medical Center, Amsterdam, The Netherlands; 7 Department of Medical Psychology, Amsterdam University Medical Centers, Meibergdreef 9, 1105AZ Amsterdam, The Netherlands; 8 Cancer Center Amsterdam (CCA), Amsterdam, The Netherlands; 9 Amsterdam Reproduction & Development Research Institute (AR&D), Amsterdam, The Netherlands

**Keywords:** Sexual functioning, Sexual satisfaction, Oncology, AYA, Young adulthood, Care delivery, Oncosexology

## Abstract

**Purpose:**

Adolescence and young adulthood (AYA) are developmental phases in which individuals establish their identity. A cancer diagnosis during this stage disrupts sexual health, but research remains limited, particularly outside of reproductive-organ cancers. This study examined perceived changes in sexual functioning from pre- to post-diagnosis among survivors and compared their current sexual functioning and satisfaction with matched controls.

**Methods:**

Dutch-speaking AYA cancer survivors (*N* = 174; age = 32.7 years; 85.6% female) recruited via convenience sampling and *N* = 348 matched controls completed self-reported measures of sexual functioning [MOS-SF] and satisfaction [GMSEX]. Paired sample *t*-tests assessed perceived changes in sexual functioning. Repeated measures ANOVAs tested the effects of sociodemographic and cancer-related factors on these variations. Independent sample *t*-tests compared current sexual functioning and satisfaction between survivors and controls.

**Results:**

Survivors reported significant declines in multiple domains of sexual functioning, including interest, arousal, orgasm function, pleasure, and lubrication (women). Greater dysfunction was observed among females, individuals diagnosed in their 30s, those in early survivorship, and those who remained with the same partner. Survivors who had engaged in partnered sexual activity reported less dysfunction than those who had not. Compared with controls, survivors reported significantly greater dysfunction and lower sexual satisfaction, while retrospectively rating their pre-diagnosis sexual functioning as more positive.

**Conclusions:**

AYA cancer survivors reported declines in sexual functioning over time, worse outcomes than controls, and more favorable recollections of their pre-diagnosis sexual health, a pattern consistent with response shift. Findings emphasize the need for comprehensive survivorship care that includes routine, proactive sexual health discussions, and tailored interventions responsive to survivors’ evolving needs.

**Supplementary Information:**

The online version contains supplementary material available at 10.1007/s00520-026-10925-2.

## Introduction

Adolescence and young adulthood represent critical developmental phases marked by physical, psychological, and psychosexual growth, during which individuals establish sexual and gender identities, form intimate relationships, and possibly pursue developmental milestones such as independence, partnerships, and parenthood [1–4]. A cancer diagnosis during this formative period profoundly disrupts these trajectories, often introducing enduring challenges to sexual health and intimacy [1, 2, 5–7]. Prevalence estimates show that 30–60% of AYA survivors report at least one domain of sexual dysfunction years after diagnosis, with variations reported by cancer type, measure, timing of assessment, and sexual activity status [1, 5, 7]. Female survivors are disproportionately affected, with 40–50% reporting dysfunction within 2 years from diagnosis [8] and more than half indicating problems with desire, arousal, or orgasm [5, 7, 9]. Common concerns include reduced sexual interest, vaginal dryness, dyspareunia, and orgasm difficulties [3, 5, 7–9]. In contrast, male survivors report somewhat lower—but still significant—rates of dysfunction, including reduced sexual satisfaction, erectile or ejaculatory difficulties, and hypogonadism-related symptoms [7, 10, 11].

Biological, treatment-related, and psychosocial factors contribute to variation in outcomes. Survivors of breast or reproductive-organ cancers (e.g., cervical and ovarian cancers) report higher dysfunction, while brain tumors and leukemia survivors frequently experience long-term psychosexual sequelae associated with cognitive and treatment-related factors [3, 6, 12]. Treatment modalities such as chemotherapy, gonadal radiation, surgery, and immunotherapy are associated with reports of greater sexual dysfunction [1–3, 8]. At the same time, psychosocial correlates, including emotional distress, body image disturbance, low social support, and relational strain, further exacerbate impairments in sexual health [5, 13].

Despite the high prevalence and distressing sequelae of cancer-related sexual dysfunction in AYAs, up to 60% of survivors report inadequate access to sexual health services or counseling, underscoring persistent gaps in survivorship care [6, 12, 14, 15]. Extant evidence highlights multiple barriers that continue to prevent AYAs from receiving adequate support, such as limited provider education, discomfort, financial constraints, and lack of specialized services [1, 4, 16, 17]. System-level failures to acknowledge or respond to survivors’ sexual health concerns contribute to increased psychological distress and reduced quality of life [14, 18]. Furthermore, a predominant focus on physical sexual dysfunction risks overlooking broader psychosocial and relational contributors such as communication barriers, caregiving dynamics, and intimacy renegotiation within couples [13–15, 19–21]. Other biases in the context of sexual health such as the neglect of unpartnered survivors or those of sexual and gender minorities contribute to additional challenges related to disclosure, dating, and sexual identity development post-cancer for these survivors [1, 3, 16, 22].

To date, most evidence investigating AYA sexual health outcomes has come from cross-sectional comparisons between survivors and healthy peers, which restricts our understanding of how sexual health concerns develop or evolve over time. [8, 9]. Furthermore, this approach neglects survivors’ subjective perceptions of change in sexual health, which are essential to understanding sexual well-being after cancer. While prospective longitudinal designs incorporating pre-diagnosis assessments would provide the most robust evidence, such studies are rarely feasible. Instead, retrospective approaches, although imperfect, provide valuable insight into perceived changes and permit the examination of treatment-related as well as developmental, contextual, and relational influences on survivorship experiences. Therefore, the present study compares AYA survivors’ perception of current sexual functioning with their recollections of pre-diagnosis experiences and benchmarks these outcomes against matched healthy controls, with the goal of characterizing perceived post-diagnosis changes and evaluating current levels of sexual functioning and satisfaction.

## Materials and methods

### Design

Data for this study are part of the *FROSA-study* (*Fertility, Romance, and Sex After Cancer in Young Adulthood* [15]), which recruited participants via online convenience sampling. Recruitment strategies included social media posts by national patient support organizations, as well as advertisements on local news outlets across the Netherlands and Belgium. The study targeted Dutch-speaking cancer survivors who were diagnosed between the ages of 18 and 39 and had completed cancer treatment. The survey was conducted anonymously, and all participants provided written informed consent online prior to beginning the survey. The local medical ethics committee of the Amsterdam UMC approved this study as exempt from in-depth review (W21_282#21.309). All procedures adhered to the principles outlined in the Declaration of Helsinki.

In total, 190 participants completed the survey, of whom 176 had finished cancer treatment. Of these, 174 participants provided complete data on the primary outcomes and were included in the current analysis. To enhance statistical power, double the number of matched controls (*N* = 348) were recruited through a commercial panel research company (Flycatcher). Controls were matched to survivors on biological sex, age, and relationship status (single or partnered) and were excluded if they had a history of cancer*.*

### Measures

Instructions explicitly recommended participants to consider any sexual or arousing activity, whether with a partner or alone. This approach broadened the scope of the selected measures, ensuring they captured both partnered and unpartnered sexual activities (i.e., masturbation, petting/making out, kissing, and oral sex). Furthermore, the utilized scales were designed to be inclusive of diverse sexual orientations, experiences, and identities, deliberately avoiding an emphasis on vaginal penetrative sex.

#### Sexual functioning [current and before diagnosis]: 

Survivors were presented with a modified 5-item version of the 4-item Medical Outcome Study-Sexual Functioning Scale (MOS-SF [23]). The MOS-SF assesses sexual interest, pleasure, arousal, and orgasm function, which we used for all participants. Additionally, we evaluated lubrication in female participants and kept the original item about erectile problems for male participants. Each sexual problem was rated on a 5-point Likert scale (*always*-*never*). Scores were standardized to a scale of 0–100 and combined into one average score, with higher scores indicating greater *dys*function [24].

These five items were presented to survivors twice. First, participants rated their *current* sexual functioning, and subsequently, they were asked to retrospectively rate their sexual functioning as they perceived it before their cancer diagnosis. Both versions were displayed on one webpage, allowing participants to adjust their responses relative to one another. Participants could also indicate if they had been sexually inactive before their diagnosis and then omitted completing the retrospective version of this scale. The MOS-SF has not been validated in Dutch, but the assessed sexual problems are also part of other more extensive and validated sexual functioning scales, including the Golombok Rust Inventory of Sexual Satisfaction (GRISS; [25]) and Female Sexual Function Index (FSFI; [26]). The item wording of the Dutch versions of the GRISS and FSFI was used in this study to ensure correct translations. Internal consistency was high, with Guttman’s lambda 0.82 (women) and 0.81 (men).

#### Sexual satisfaction [current]: 

The 5-item Global Measure of Sexual Satisfaction (GMSEX, [27]) uses bipolar anchors to describe general sexual satisfaction (e.g., *good-bad*, *comfortable-uncomfortable*). Between those anchors, a 7-point Likert scale is presented to participants to indicate how they perceive their sex lives. Scores were standardized to range between 0 and 100. Given the simplicity of answer options, these were translated by the research team. GMSEX showed high reliability in this sample (*α* = 0.94 for women and 0.92 for men).

### Statistical analysis

Data was analyzed using SPSS Statistics, version 28. Descriptive statistics were used to summarize participant responses. Current sexual functioning was reported and analyzed for differences based on sociodemographic factors (sex, age, relationship status, partnered sexual activity) using *t*-tests and Pearson’s correlation respectively. Descriptive statistics were also reported for each of the five individual sexual functioning items. Associations between sexual functioning and satisfaction were tested using Pearson’s correlation coefficient.

Perceived changes in sexual functioning were tested using paired sample *t*-tests (for the composite score and separate items). Subsequently, the effects of sociodemographic and cancer-related factors on such change were assessed using repeated measures ANOVAs. Tested factors included biological sex, changed relationship status since diagnosis (same partner/changed), partnered sexual activity in the past four weeks, age at diagnosis (twenties or younger vs. thirties; controlling for current age), phase of survivorship determined by years since diagnosis (i.e., very short term (< 1 year), short term (1–5 years), long term (5+ years) survivorship), type of diagnosis, and primary treatment modalities (surgery, chemo, radiation). Each of these factors was tested in separate repeated measures ANOVA to test their relative contribution.

Finally, current sexual functioning and satisfaction were compared to healthy controls, using independent sample *t*-tests. Significance testing was accompanied by calculating Hedge’s *g* effect sizes to estimate the magnitude of differences. Hedge’s *g* is interpreted like Cohen’s *d* (*g* ≥ 0.2 is small, ≥ 0.5 moderate, ≥ 0.8 large), but uses a correction to prevent overestimation [28].

## Results

### Participants

Survivors (*N* = 174) were predominantly female (*n* = 149, 85.6%), had a mean age of 32.7 years (SD = 6.1, range = 20–53 years, median =33.0 years), and were 0–24 years from diagnosis. Age at first diagnosis ranged from 12 to 39 years, with 42.0% having been diagnosed in their 30s. The most common types of cancer diagnoses were breast cancer (*n* = 53, 30.5%) and leukemia/lymphoma (*n* = 52, 29.9%). Approximately three quarters of survivors were partnered (*n* = 131, 75.3%), of which 101 (58.0%) were still with the same partner since their cancer diagnosis. Two-thirds (*n* = 109, 62.6%) reported engaging in partnered sexual activities in the 4 weeks prior to study participation. The matched healthy control group (*N* = 348) was also 85.6% female, with a mean age of 32.7 years (range: 19–54 years), and 75.3% were partnered (Table 1).

**Table 1:** Descriptive statistics of all participants

	Survivors (*N* = 174)		Controls (*N* = 348)	
**Age in years**, M (SD), range	32.7 (6.1), 20–53		32.7 (6.3), 19–54	
	*n*	%	*n*	%
**Sex**				
Female	149	85.6%	298	85.6%
Male	25	14.4%	50	14.4%
**Relationship status**				
Single	43	24.7%	86	24.7%
Partnered	131	75.3%	262	75.3%
**Relationship status change since diagnosis**				
Same partner	101	58.0%		
Change in partner/opportunity to date	73	42.0%		
Partnered sexual activity in previous 4 weeks*				
Yes	109	62.6%		
No	55	31.6%		
**Age at diagnosis**				
≤ 29	101	58.0%		
≥ 30	73	42.0%		
**Survivorship phase**				
Very short term (< 1 year)	73	42.0%		
Short term (1–5 years)	66	37.9%		
Long term (5+ years)	35	20.1%		
**Type of diagnosis**				
Breast cancer	53	30.5%		
Leukemia/lymphoma	52	29.9%		
Gynecological cancers	27	15.5%		
Testicular cancer	11	6.3%		
Other**	31	17.8%		
**Primary types of treatment combinations**				
Surgery only	24	13.8%		
Surgery + chemotherapy	30	17.2%		
Surgery + chemotherapy + radiotherapy	43	24.7%		
Chemotherapy only	20	11.5%		
Chemotherapy + radiotherapy	22	12.6%		
Surgery + chemotherapy + radiotherapy + immunotherapy	12	6.9%		
Other combinations	23	13.2%		

*Missing data for partnered sexual activity: *n* = 10 (5.8%)

**Excluded from analyses involving the type of diagnosis

### Current sexual functioning and satisfaction

Survivors reported an average sexual dysfunction score of *M* = 48.0, with substantial variability of scores ranging between 0 and 95. These scores correspond to 17.8% of survivors reporting no/minimal dysfunction (scores ≤ 25), 39.1% reporting mild dysfunction (scores 26–50), 35.1% reporting moderate dysfunction (scores 51–75), and 8.0% reporting severe dysfunction (scores ≥ 76). At item level, sexual problems that were most often endorsed were loss of sexual interest (51.7% reported “never” or “occasionally” interest) and arousal (40.8% reported “never” or “occasionally”; see Appendix-1).

When testing sociodemographic factors, significant differences were detected between male (*n* = 25) and female survivors (*n* = 149). Female respondents reported substantially higher sexual dysfunction than males (*M* = 51.0 vs. 30.2; *t*_(172)_ = 4.80, *p* < 0.001, *g* = 1.03), though the small male subsample limits generalizability. Sexual functioning did not differ by relationship status (*M* = 46.4 for single vs. 48.5 for partnered survivors; *t*_(172)_ = 0.564, *p* = 0.573, *g* = 0.10). Instead, it differed by whether survivors had engaged in partnered sexual activities. Those who had engaged in activities with a partner (*n* = 109) reported less dysfunction than those who were inactive (*n* = 55; *M* = 43.6 vs. 58.1; *t*_(162)_ = 4.37, *p* < 0.001; *g* = 0.72). Notably, 26.8% of single survivors reported having been sexually active, while 20.3% of partnered survivors reported sexual inactivity within the same period. Sexual dysfunction was unrelated to participants’ current age (*r* = 0.019, *p* = 0.802). Survivors’ current sexual satisfaction had a mean score of *M* = 63.1 (range = 0–100), and it was moderately and negatively correlated with sexual dysfunction (*r* = −0.585, *p* < 0.001).

### Perceived changes in sexual functioning

Four survivors reported no sexual experiences before their cancer diagnosis, and another eight reported experiences but did not respond to all assessed items. This left *n* = 162 participants (87.4%) with complete data on retrospective sexual experiences for analyses. Compared to their pre-cancer perceived sexual function, survivors reported a significant worsening after diagnosis (*M* = 25.4 vs. 48.2; *t*_(161)_ = 12.06, *p* < 0.001, *g* = 0.94). This self-reported deterioration was observed across all sexual problem items (all *p* < 0.001, *g* > 0.5), except for erectile dysfunction in men (see Table 2).

**Table 2:** Comparison of sexual functioning problems: current vs. recalled pre-diagnosis, and relative to controls

	AYA survivors (*N* = 162) *			AYA survivors (*N* = 174)	Controls (*N* = 341)	
	Pre-cancer [pre-diagnosis]	Current [post-treatment]	Comparison between time points	Current [post-treatment]	Current	Comparison between AYAs and controls
	M (SD)	M (SD)	*t* (*df*), *p*, *g*	M (SD)	M (SD)	*t* (*df*), *p*, *g*
Total score MOS-SF	25.4 (17.0)	48.2 (21.4)	12.06 (161), *p* <.001, *g* = 0.94	48.0 (21.3)	40.3 (19.6)	4.07 (513), *p* <.001, *g* = 0.38
Single items:						
Sexual interest	35.2 (19.8)	62.0 (23.0)	12.82 (161), *p* <.001, *g* = 1.00	61.2 (22.9)	54.0 (23.1)	3.37 (513), *p* <.001, *g* = 0.31
Sexual pleasure	23.1 (21.7)	39.8 (27.2)	6.58 (161), *p* <.001, *g* = 0.51	39.7 (27.3)	33.1(25.2)	2.70 (513), *p* =.004, *g* = 0.25
Sexual arousal	22.8 (20.4)	51.4 (27.2)	11.36 (161), *p* <.001, *g* = 0.89	50.7 (27.1)	45.8 (26.0)	2.02 (513), *p* =.022, *g* = 0.19
Orgasm	30.6 (27.2)	44.4 (30.3)	7.18 (161), *p* <.001, *g* = 0.56	45.3 (30.2)	40.2 (28.8)	1.86 (513), *p* =.032, *g* = 0.17
Erectile dysfunction (men)	9.8 (12.5)^a^	16.3 (22.1)^a^	1.66 (22), *p* =.110, *g* = 0.34	19.0 (24.2)^c^	7.5 (13.6)^c^	2.21 (73), *p* =.030, *g* = 0.64
Lubrication (women)	16.2 (20.8)^b^	48.0 (29.7)^b^	11.16 (138), *p* <.001, *g* = 0.94	47.2 (29.6)^d^	32.2 (24.5)^d^	(438), *p* <.001, *g* = 0.57
Total score GMSEX	**-**	**-**		63.1 (25.8)	70.8 (22.5)	−3.47 (513), *p* <.001, *g* = 0.32

See Appendix-1 for descriptive statistics of all response categories for each item

*All survivors with complete data on both current and pre-cancer sexual functioning

^a^*n* = 23 male survivors and 46 male controls

^b^*n* = 139 female survivors and 278 female controls

^c^*n* = 25 male survivors and 50 male controls

^*d*^*n* = 149 female survivors and 291 female controls

Separate repeated measures ANOVA was used to assess which factors were associated with perceived changes. Inherently, all repeated measures ANOVAs reiterated the perceived increase in sexual dysfunction over time. Moreover, it was indicated that male and female survivors significantly differed from each other, while the interaction effect of sex and time was only borderline significant (*F*_(1, 160)_ = 3.74, *p* = 0.055). This suggests that women reported higher sexual dysfunction overall, while an increase in perceived sexual dysfunction was somewhat steeper over time in female than male survivors (see estimated marginal means, *EMM* in Appendix-2).

**Fig. 1:**
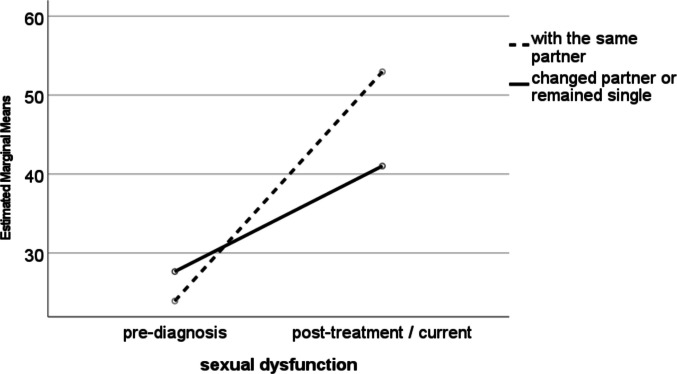
Perceived changes in sexual functioning from pre-diagnosis to current (post-treatment) by whether survivors’ relationship status changed

Second, perceived changes in sexual dysfunction were strongly associated with changes in relationship status: (*F*_(1,160)_ = 18.09, *p* < 0.001) survivors who were still with the same partner reported a much more substantial increase in sexual dysfunction than those who had new partners or dating experiences (see *EMMs* in Fig. 1).

**Fig. 2:**
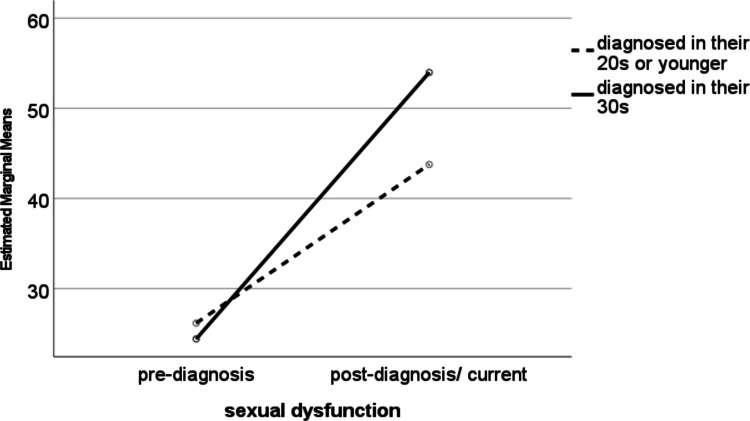
Perceived changes in sexual functioning from pre-diagnosis to current (post-treatment) by age at cancer diagnosis

Third, the possible effects of age at diagnosis were tested, controlling for current age. Current age was not significant (*F*_(1,159)_ = 2.61, *p* = 0.109), while age at diagnosis showed significant effects (*F*_(1,159)_ = 7.93, *p* = 0.005). Survivors who had been diagnosed in their 30 s reported steeper increases in sexual dysfunction relative to those diagnosed in their 20 s (see *EMMs* in Fig. 2).

**Fig. 3:**
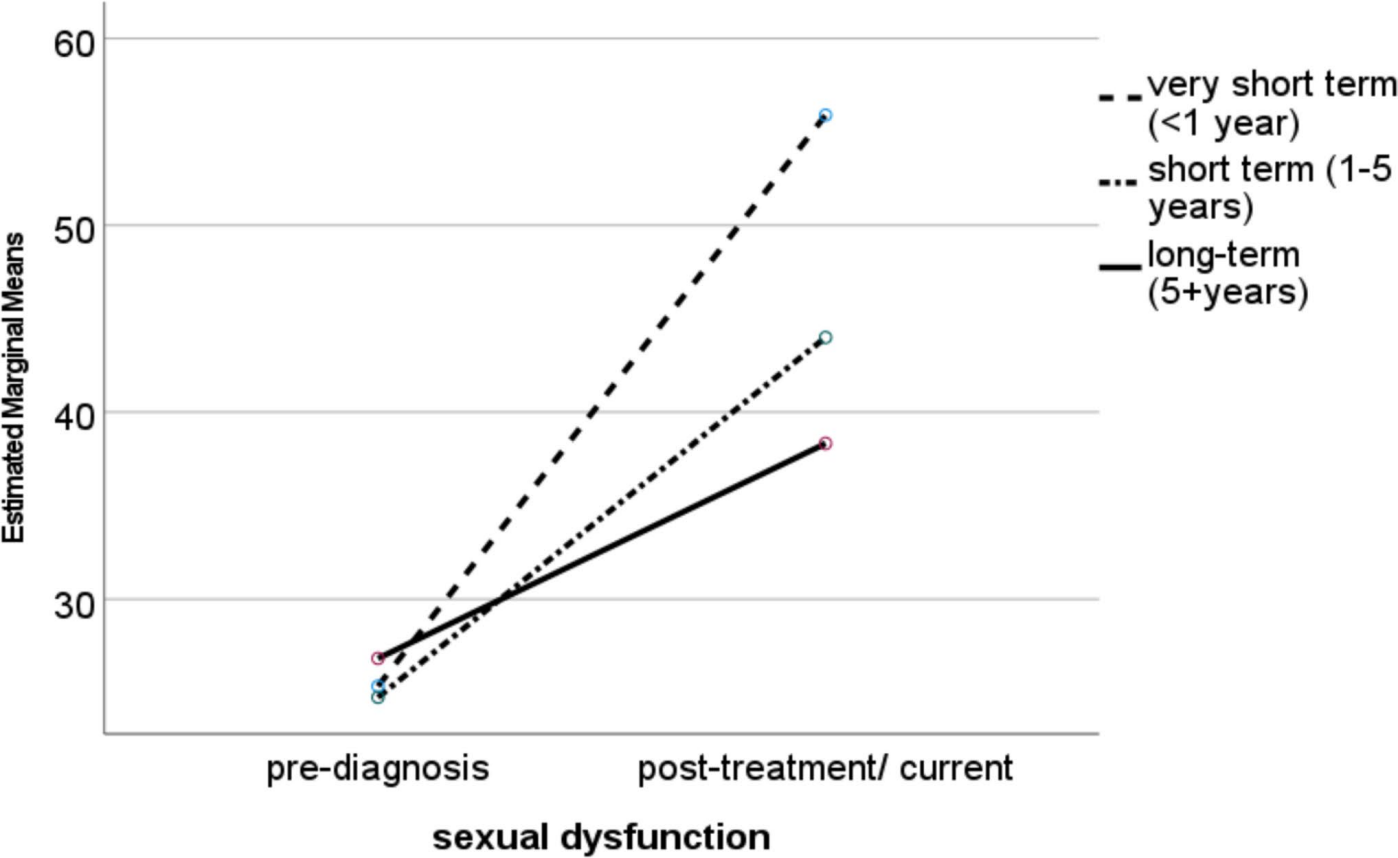
Perceived changes in sexual functioning from pre-diagnosis to current (post-treatment) by survivorship phase

Fourth, survivors who were in different phases of survivorship reported diverse sexual functioning (*F*_(2,159)_ = 4.44, *p* = 0.013) and these scores changed differently over time (*F*_(2,159)_ = 8.37, *p* < 0.001). In other words, survivors who were still in their first year since diagnosis reported the steepest increase in sexual dysfunction relative to those diagnosed between 1 and 5 years (i.e., short term) and 5+ years ago (i.e., long term; see EMMs in Fig. 3).

Fifth, the type of diagnosis was tested comparing breast cancer, leukemia/lymphoma, gynecological cancers, and testicular cancer (i.e., excluding “other” types of diagnosis to allow for more meaningful comparisons). Sexual dysfunction significantly differed between diagnostic groups (*F*_(3,130)_ = 6.22, *p* < 0.001) but did not change as a function of cancer type over time (i.e., no significant interaction effect (*F*_(3,130)_ = 2.11, *p* = 0.102). All groups reported similar levels of sexual functioning before diagnosis and all deteriorated. Yet, current dysfunction was lowest in testicular cancer survivors relative to all other diagnostic groups, and lower in leukemia/lymphoma survivors relative to breast cancer survivors (see EMMs in Appendix-3).

Sixth, different combinations of primary treatment modalities (see also Table 1) did not show significant effects on changes in sexual functioning between the two time points (*F*_(4,126)_ = 1.91, *p* = 0.114), but larger samples are needed to robustly test all possible treatment combinations (see Appendix-4). Interestingly, 30 participants (17.2%) reported the ongoing use of adjuvant hormone therapy. Exploratory analyses indicated that survivors receiving hormone therapy reported similar pre-diagnosis sexual functioning but lower post-treatment sexual functioning than survivors not receiving hormone therapy. Accordingly, they reported a steeper increase in sexual dysfunction from pre-diagnosis to post-treatment, suggesting a decline in sexual functioning over time (see Appendix-5).

Notably, using the Bonferroni correction and adjusting the *p*-level to *p* = 0.008 (due to multiple testing with six separate repeated measures ANOVA), all three of the above significant interaction effects are still considered significant: perceived sexual dysfunction significantly worsened over time as a function of relationship status change, age at diagnosis, and survivorship phase (see Figs. 1, 2, 3).

### Current sexual functioning and satisfaction relative to healthy controls

Compared to matched controls, AYA cancer survivors reported significantly greater sexual dysfunction (*t*_(513)_ = 4.07, *p* < 0.001), which constitutes a rather small difference (*g* = 0.38; *M* = 48.0 vs. 40.3 respectively). At item level, it appeared that this difference was salient for sexual interest (*t*_(513)_ = 3.37, *p* < 0.001; *g* = 0.31) and pleasure (*t*_(513)_ = 2.70, *p* = 0.009; *g* = 0.25), but not arousal or orgasm function (*p* > 0.05; *g* < 0.188). More pronounced differences were observed on the two sex-specific items: male survivors reported considerably worse erectile dysfunction (*g* = 0.64) while female survivors experienced worse lubrication (*g* = 0.57) than healthy controls respectively. Survivors also reported somewhat lower sexual satisfaction than controls (*M* = 63.1 vs. 70.8 respectively, *t*_(511)_ = 3.47, *p* < 0.001; *g* = 0.32). Interestingly, survivors’ sexual functioning as perceived before diagnosis (i.e., in a healthy state) was remembered more positively than the current sexual functioning of healthy controls (see Appendix-6).

## Discussion

This study provides a comprehensive analysis of perceived sexual functioning among AYA survivors, revealing significant differences compared to matched healthy controls and elucidating perceived changes over time and the factors influencing such changes. Notably, cancer survivors retrospectively rated their pre-diagnosis sexual functioning more positively than healthy controls currently, which indicates possible response shift being at play. We return to this finding later in the discussion.

Over 80% of participants (82.2%) reported at least mild sexual dysfunction, with loss of sexual interest and arousal difficulties being most common concerns in the sample. Sexual satisfaction, while moderately correlated with dysfunction, was higher than expected given survivors’ levels of impairment, suggesting complex adaptive processes at play. Although sexual satisfaction and sexual function are related constructs, they are not interchangeable. Sexual satisfaction reflects a broader evaluation of one’s sexual life and may be influenced by emotional intimacy, relationship quality, communication, and partner responsiveness. Consequently, individuals may experience substantial changes in physical sexual function while maintaining relatively positive evaluations of their sexual intimate relationships. As survivors often redefine sexual intimacy, place greater value on non-sexual aspects of closeness, or adjust expectations regarding sexual activity, such aspects of satisfaction may become more salient. These findings are consistent with biopsychosocial models of sexual health and extend prior research documenting persistent and multidimensional sexual health concerns in AYA survivorship [1, 3, 5, 6, 8, 9, 12, 29].

Consistent with existing literature, sex differences were pronounced. Female survivors reported substantially greater dysfunction, particularly in domains of desire, arousal, lubrication, and orgasm, while male survivors, though underrepresented, also reported difficulties such as erectile and ejaculatory dysfunction [4, 5, 9–11, 16]. These findings highlight the gendered nature of sexual late effects but also underscore that dysfunction is not exclusively female, reinforcing the importance of sex-specific and inclusive approaches to assessment and care [10, 16].

Engagement in partnered sexual activity was associated with lower dysfunction regardless of relationship status, which appears to show that positive sexual experiences (i.e., less sexual problems) may reinforce partnered activity and vice versa [16, 30]. Yet, survivors who remained with the same partner since diagnosis reported greater deterioration, echoing evidence that couples often struggle to renegotiate intimacy in the face of grief over pre-cancer sexuality, physical changes, and caregiving dynamics [19, 31]. Relational coping processes, such as disclosure, communication, and shared problem-solving strategies, are critical for sexual recovery and should be central targets for dyadic interventions [30, 32].

Healthcare providers frequently neglect discussions about sexuality with cancer patients and survivors [17, 22, 29, 33–35], particularly among unpartnered individuals, reflecting biases that assume singles are less sexually active. Furthermore, clinical care often relies on heteronormative definitions of sexuality that prioritize penetrative (vaginal) sex, neglecting diverse forms of sexual expression such as masturbation, pleasure, and physical intimacy [34, 35]. This limited perspective fails to account for the broader sexual health needs of AYA patients and survivors and may contribute to our finding that unpartnered survivors appear particularly vulnerable, as they navigate disclosure, dating, and establish their sexual identity in the context of cancer survivorship without access to the same amount of suitable information and resources.

Outcomes also varied by age at diagnosis: AYA survivors diagnosed in their 30 s reported steeper declines than those diagnosed younger, likely to reflect more pervasive disruption of developmental milestones and established sexual repertoires [5, 16, 21]. Such developmental tasks include, for example, long-term partnership formation, reproductive decision-making, or parenting. In addition, individuals in their 30 s may have had more time to establish a stable sexual identity, develop preferences and expectations regarding sexual activity, and have cultivated a familiar sexual repertoire. Consequently, cancer-related changes in sexual function may be experienced as a more substantial disruption to an established sense of sexual self and sexual preferences. In contrast, younger survivors may still be actively exploring aspects of sexuality, relationships, and identity, potentially influencing how they perceive and adapt to sexual changes following cancer. Future research is needed to better understand how developmental stage and sexual self-concept shape survivors’ experiences of sexual adjustment after cancer. Finally, outcomes also varied by survivorship phase showing that those within the first-year post-treatment described the greatest deterioration, consistent with prior studies showing heightened vulnerability for impaired sexual function in early survivorship [1, 8].

A key finding from the present work, with implications for clinical care, entails that AYA survivors retrospectively rated their pre-diagnosis sexual health more positively than healthy controls. This was an unexpected result that cannot be explained by cancer-related treatment effects alone. This pattern is consistent with response shift theory, whereby individuals recalibrate internal standards and reframe expectations in the wake of illness [36–38]. Survivors may perceive losses more acutely when comparing current functioning to a possibly idealized pre-cancer baseline; yet they also tend to adjust their satisfaction with sexual relationships by redefining what constitutes acceptable or meaningful sexual health. This dual process helps explain why moderate dysfunction co-exists with higher-than-expected satisfaction in our sample. Utilizing response shift as a possible explanatory mechanism clarifies the link between measured quantitative changes in self-reported sexual function, survivors’ evolving cognitive appraisals, and lived experiences of sexuality and sexual well-being. This also suggests that mixed methods and community-engaged approaches are needed to better study and understand these dynamics.

### Clinical implications

There is an urgent need to better integrate sexual health discussions and assessments into routine AYA acute and survivorship care. From a response-shift perspective, perceived declines in sexual functioning may reflect not only changes in function itself but also changes in the priorities, expectations, and reference points individuals use to evaluate their sexual experiences. Acknowledging this possibility requires a careful balance between validating survivors’ experiences of grief, loss, and distress and recognizing that cancer may fundamentally alter how individuals understand and appraise their sexual lives. Rather than questioning the validity of retrospective evaluations, this perspective invites consideration of how shifts in meaning, values, and expectations may shape perceptions of change over time. Such an understanding may enrich clinical conversations and help identify previously valued sources of intimacy, pleasure, and sexual well. At the same time, these findings also highlight that addressing sexual health from the time of diagnosis onward would help ensure that patients receive early identification of concerns and that they can access ongoing support, including referrals to specialized sex therapy and counseling when needed. It is essential to adopt a comprehensive, multidisciplinary approach grounded in a biopsychosocial model [2, 20] that addresses the interconnected physical, emotional, cultural, and relational dimensions of sexuality. Such an approach should be tailored to each survivor’s age, sex, cancer type, relationship status, and sexual identity/orientation. Relationship-focused interventions, such as communication skills training and intimacy enhancement, can help AYA survivors and their partners navigate sexual losses and rediscover pleasurable and satisfying forms of sexual intimacy [39, 40]. At the same time, interventions must also address the distinct challenges faced by single survivors approaching dating, disclosure, and sexual activities. Programs that build self-esteem, normalize diverse forms of sexual expression, and provide safe opportunities for survivors to reclaim their sexual identity are crucial.

### Limitations

Although this contribution offers novel insights into perceived changes in sexual functioning and satisfaction with sexual relationships, several limitations warrant consideration. First, online convenience sampling and reliance on retrospective self-report data may introduce selection and recall bias. Second, the small male subsample limits the generalizability of sex-specific findings, underscoring the need for larger, more diverse samples in future studies. Third, the absence of multimethod assessments (e.g., hormonal profiles, validated clinical measures) constrains the ability to triangulate self-reported outcomes. Longitudinal designs that capture trajectories of sexual health over time would further clarify patterns of loss, adaptation, and recovery, as well as inform the optimal timing of interventions. Finally, greater attention to identity-level, cultural, relational, and socioeconomic factors is needed to ensure that future interventions are inclusive and contextually tailored. Despite these limitations, the strengths of this study include the use of a large, well-matched control group and rigorous statistical adjustments, which provide confidence in the robustness of our findings.

## Conclusion

The present study highlights the profound and complex impact of cancer on the sexual health of AYA cancer survivors. Marked declines in sexual functioning and satisfaction relative to both healthy peers and survivors’ own pre-diagnosis appraisal highlight the unique vulnerabilities of this population, with particularly pronounced effects in subgroups defined by sex, age, relationship status change, or survivorship phase. These findings emphasize the importance of initiating sexual health interventions early across the cancer continuum and delivering them through tailored, multidisciplinary approaches. Future research should prioritize longitudinal mixed-method designs with diverse, sexual, and gender-inclusive samples, incorporating partner perspectives where applicable, and explicitly situate findings within survivors’ developmental and relational contexts.

## Supplementary Information

Below is the link to the electronic supplementary material.

ESM1 (PDF 467 KB)

## Data Availability

The datasets generated and/or analyzed during the current study are available from the corresponding author on reasonable request.
