# Supplementary material for: Appraisal of sexual functioning before and after cancer: insights from adolescent and young adult (AYA) cancer survivors and matched healthy controls
Source: Support Care Cancer. 2026 Jun 25;34(7):687. doi: 10.1007/s00520-026-10925-2 (PMC13294235; doi:10.1007/s00520-026-10925-2)
Supplement: Supplementary file 1 — (PDF 467 KB) [file 520_2026_10925_MOESM1_ESM.pdf]

APPENDIX TO THE MANUSCRIPT: ***Appraisal of Sexual Functioning Before and After Cancer: Insights from Adolescent and Young Adult (AYA) Cancer Survivors and Matched Healthy Controls***

Chiara Acquati, Brenda L. den Oudsten, Stephanie Both, & Vicky Lehmann

Contact:
v.lehmann@amsterdamumc.nl or v.lehmann@pl.hanze.nl

Data are part of the FROSA-study:
FROSA = **F**ertility, **RO**mance, and **S**ex in Young **A**dulthood

Overview

Appendix-1: Percentages of endorsed sexual problems page 1
Appendix-2: Perceived changes in sexual functioning by sex page 2
Appendix-3: Perceived changes in sexual functioning by type of diagnosis page 2
Appendix-4: Perceived changes in sexual functioning by primary treatment page 3
Appendix-5: Perceived changes in sexual functioning by hormone therapy use page 4
Appendix-6: Box Plot sexual dysfunction survivors vs. controls page 5

Appendix-1

Percentages of endorsed sexual problems (MOS-items) before and after treatment

higher score = greater problem	**Always** (1)	**Most of the time** (2)	**Regularly** (3)	**Occasionally** (4)	**Never** (5)
**Sexual Interest**					
Pre-diagnosis	11.1%	45.1%	35.8%	8.0%	-
Post-treatment [currently]	1.7%	13.8%	32.8%	41.4%	10.3%
**Sexual Pleasure**					
Pre-diagnosis	34.6%	45.1%	13.6%	6.8%	-
Post-treatment [currently]	15.5%	39.7%	17.8%	24.7%	2.3%
**Sexual Arousal**					
Pre-diagnosis	32.1%	50.0%	12.3%	5.6%	-
Post-treatment [currently]	7.5%	28.7%	23.0%	35.1%	5.7%
**Orgasm Function**					
Pre-diagnosis	28.4%	40.1%	14.8%	14.2%	2.5%
Post-treatment [currently]	15.5%	31.0%	16.7%	30.5%	6.3%
**Erectile Function (men)**					
Pre-diagnosis	60.9%	39.1%	-	-	-
Post-treatment [currently]	48.0%	40.0%	-	12.0%	-
**Lubrication Function (women)**					
Pre-diagnosis	54.0%	31.7%	10.1%	4.3%	-
Post-treatment [currently]	14.1%	28.9%	16.1%	36.2%	4.7%

Appendix-2

Perceived changes in sexual functioning from pre-diagnosis to current (post-treatment) by sex

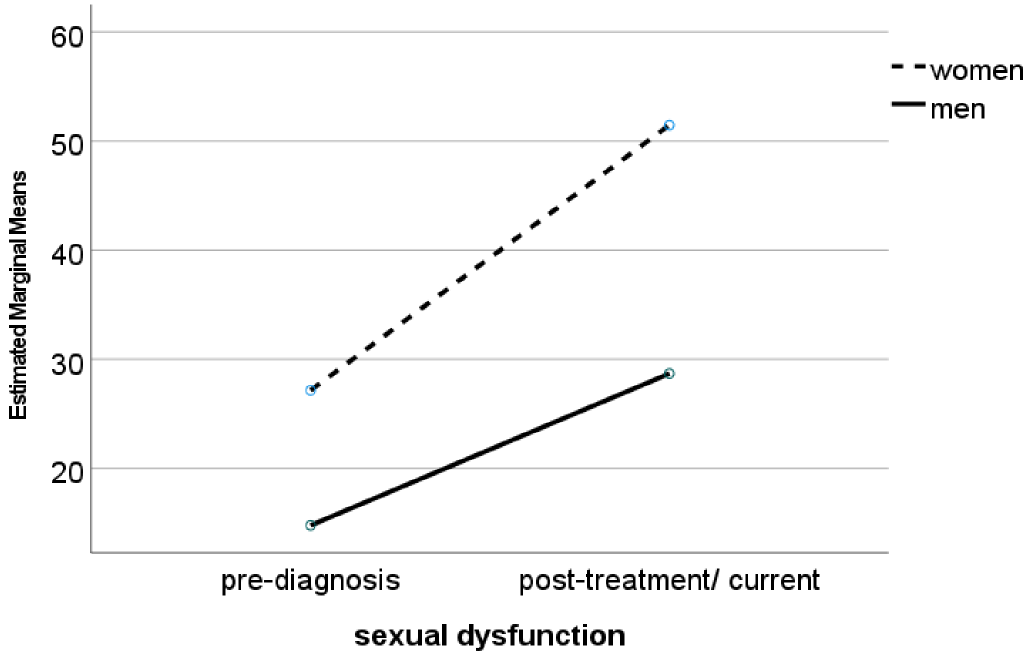

Appendix-3:

Perceived changes in sexual functioning from pre-diagnosis to (current) post-treatment by type of diagnosis

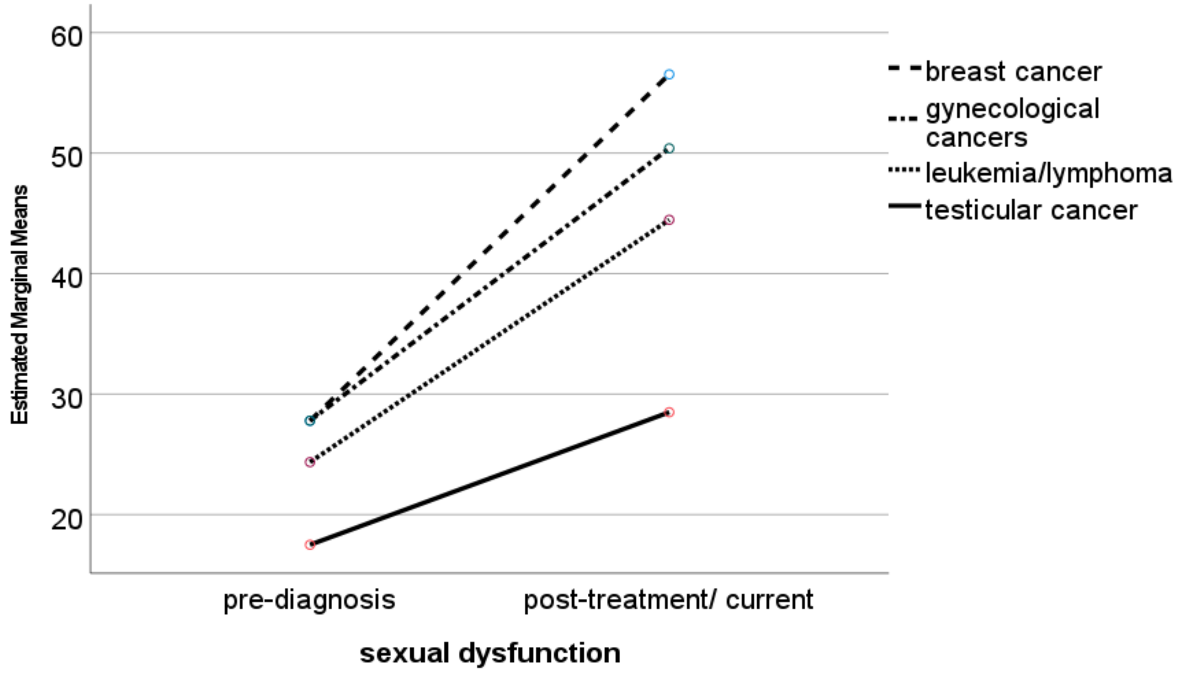

Appendix-4:

Perceived changes in sexual functioning from pre-diagnosis to (current) post-treatment by primary treatment combinations. This included:

Surgery only	24 (13.8%)
Surgery + chemotherapy	30 (17.2%)
Surgery + chemotherapy + radiotherapy	43 (24.7%)
Chemotherapy only	20 (11.5%)
Chemotherapy + radiotherapy	22 (12.6%)

Note: other types of treatment combinations were omitted due to low numbers

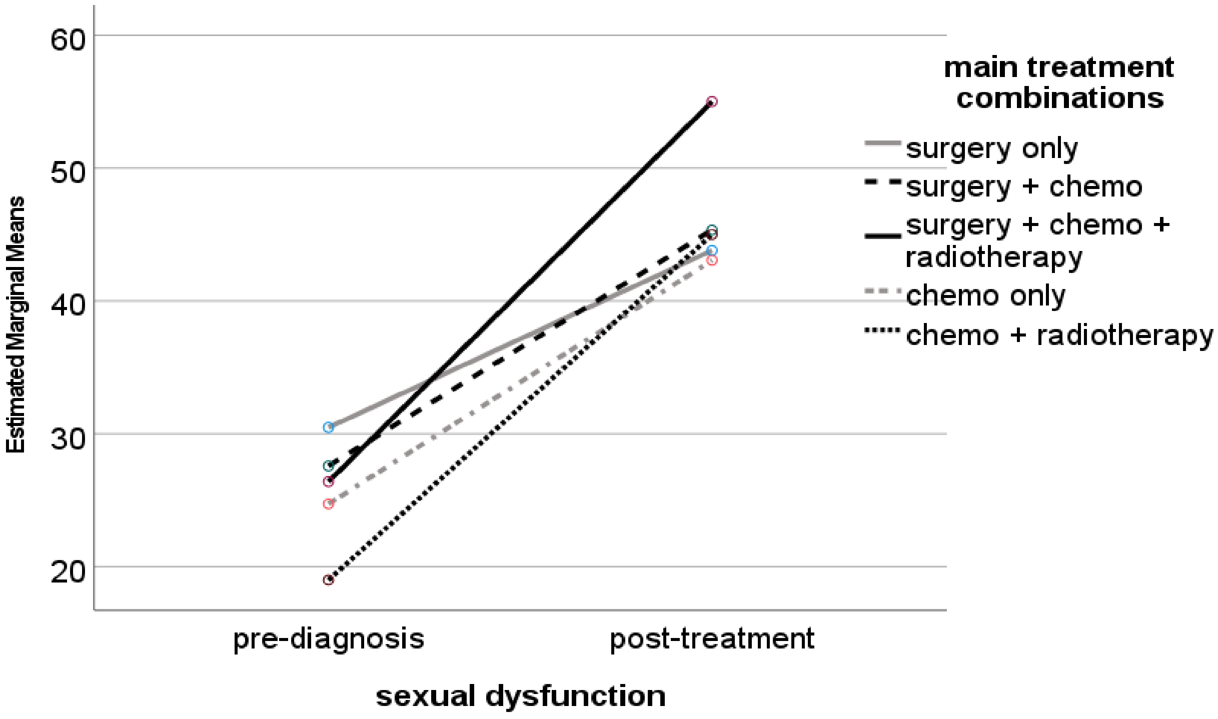

Appendix-5:

Perceived changes in sexual functioning from pre-diagnosis to (current) post-treatment by adjuvant hormone therapy use

Note: n=30 reported ongoing hormone therapy use, all of whom were female and almost all were breast cancer survivors (n=28)

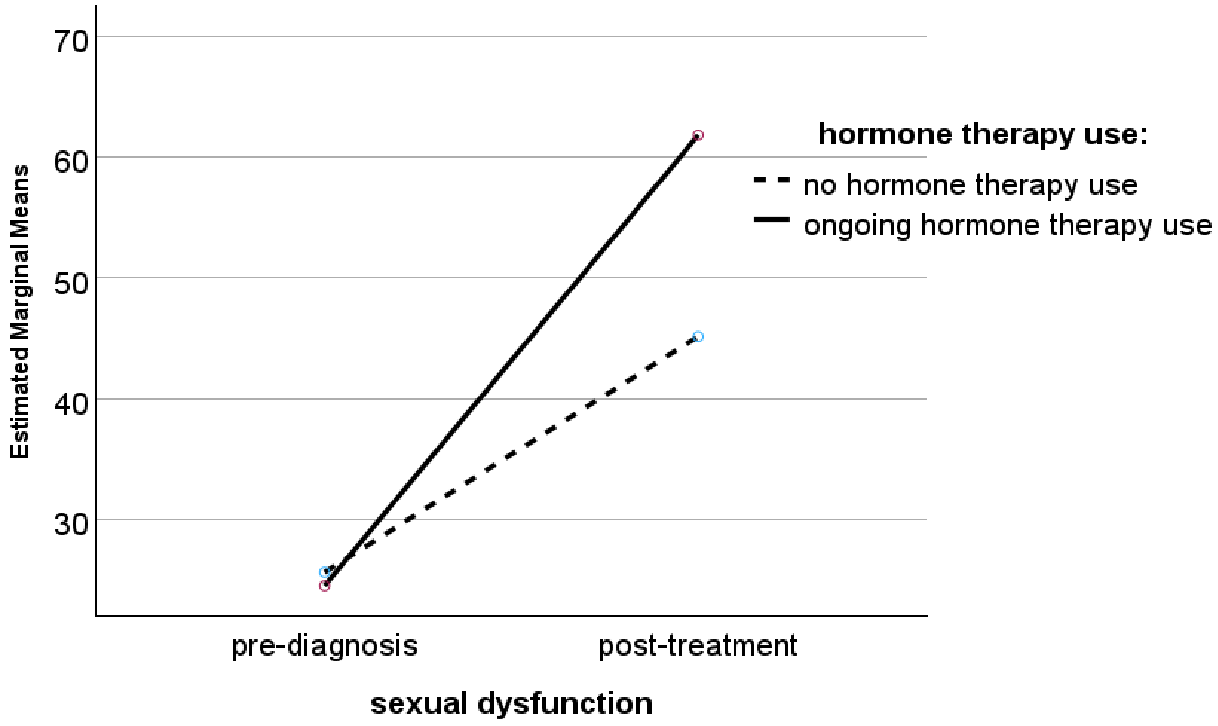

Appendix-6:

Box Plot (means, percentiles, median, minimum, and maximum) of observed scores of sexual dysfunction for survivors pre-diagnosis (green), controls currently (grey), and survivors currently (orange).

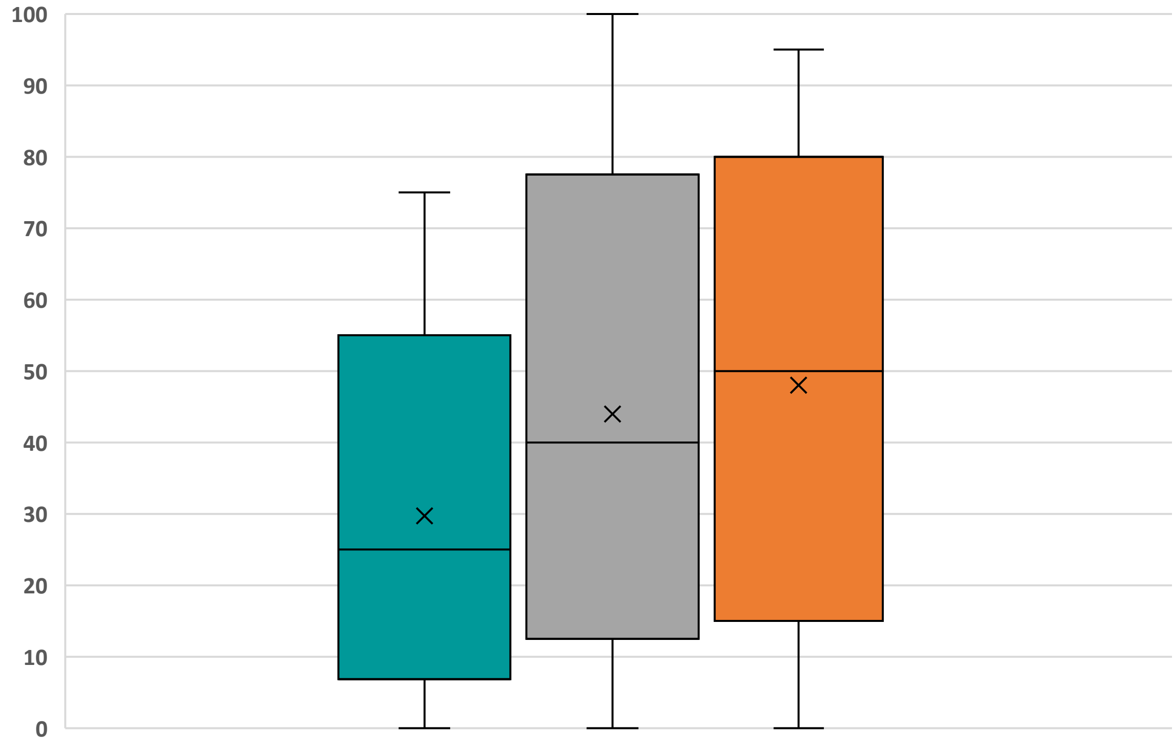

**Higher scores indicate greater dysfunction*
